# A Tailored Approach to the Ablation of Atrial Fibrillation in Rheumatic Valvular Disease with High-density Grid Technology

**DOI:** 10.19102/icrm.2021.120120S

**Published:** 2021-01-15

**Authors:** Mariano Rillo, Zefferino Palamà, Giulia My, Raffaele Punzi, Andrea Aurelio, Angelo Aloisio, Francesco Zonno, Cesare Giannattasio, Luigi My

**Affiliations:** ^1^Electrophysiology Service, Division of Cardiology – Casa di Cura Villa Verde, Taranto, Italy; ^2^Cardiology Unit – Casa di Cura “Villa Verde,” Taranto, Italy; ^3^Abbott Medical Italia, Sesto San Giovanni, Italy

**Keywords:** Ablation, Advisor HD Grid, atrial fibrillation, high-density mapping

Atrial fibrillation (AF) ablation can be particularly challenging in the setting of valvular rheumatic disease due to the organic structural alterations of the left atrium (LA) inherent with this condition. The use of high-density (HD) maps can improve the operator’s ability to target the arrhythmia with a tailored approach, especially in complex cases.

We report the case of a 64-year-old female patient with rheumatic severe mitral regurgitation treated by mitral valve prosthetic ring annuloplasty. We performed AF ablation using the EnSite™ Precision™ mapping system; the multipolar Advisor™ HD Grid Mapping Catheter, Sensor Enabled™; and the EnSite™ LiveView software tool, which allows dynamic “beat-to-beat” activation and voltage-mapping visualization. HD mapping (39.070 points) performed during AF showed low voltage (LV) spread throughout the entire LA, so we conducted synchronized electrical cardioversion. The new HD map (26.821 points) was completely different, showing some areas of the anterior and septal walls with voltages of greater than 0.6 mV (purple color indicating normal tissue in **Figure 1**) alternating with areas of LV of less than 0.3 mV (grey color indicating scar tissue in **Figure 1**), while the remaining areas of the LA were mostly healthy (purple color). We performed complete posterior vein isolation and posterior wall isolation **(Video 1)**. No lesions were created in the remaining anatomical region in order to leave as much healthy tissue as possible. At five months of follow-up, no AF recurrence was documented.

In our case, we constructed very HD maps (> 25,000 points acquired) using omnipolar technology; this approach proved to be insensitive to catheter orientation unlike the bipolar mapping and supported us in the attempt to perform a “true tailored” ablation, helping us to save the anatomical areas characterized by healthy tissue and focus on only the substrates that probably would not be identified with bipolar HD mapping. HD mapping with the use of an omnipolar catheter, especially in a very complex scenario like rheumatic valvular disease, represents a fundamental tool with which to precisely search AF substrates and tailor the ablation strategy.

## Figures and Tables

**Figure 1:**
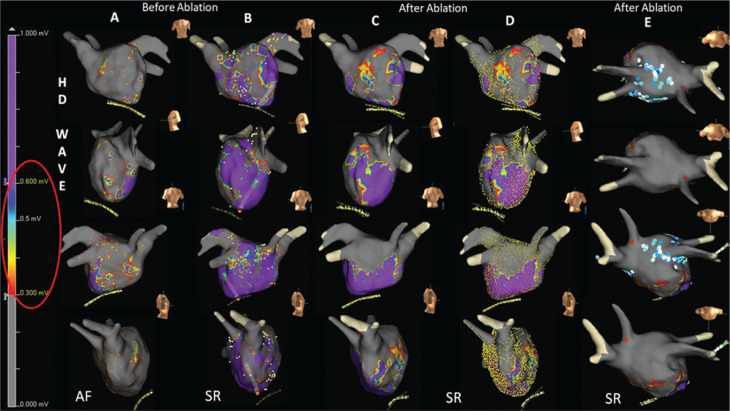
LA HD voltage maps acquired using the multipolar Advisor™ HD Grid Mapping Catheter, Sensor Enabled™ and the EnSite™ Precision™ mapping system in an HD-wave configuration using the voltage range of 0.3 to 0.6 mV. **A:** Map recorded before ablation with 39.070 points acquired during AF showing an LV of less than 0.3 mV that affected most of the LA, compatible with scar tissue (grey color). **B:** Map recorded before ablation during sinus rhythm showing the prevalence of voltages of greater than 0.6 mV (purple color), compatible with normal tissue in most of the LA. **C:** Map recorded after ablation demonstrating the strategy adopted to treat only areas of the posterior and anterior walls of the LA presenting LV in the range of 0.3 to 0.6 mV, as a set of different colors, possibly indicative of patchy fibrosis. **D:** Maps showing the number of points acquired (> 25,000), evenly distributed over the entire left atrial volume. **E:** Cranial projections of the map after ablation clearly demonstrating complete isolation of the pulmonary veins and posterior wall.

**Video 1.** EnSite™ LiveView activation map postablation showing pulmonary vein and posterior wall isolation.

